# Synthesis and Evaluation of ^177^Lu-DOTA-DN(PTX)-BN for Selective and Concomitant Radio and Drug—Therapeutic Effect on Breast Cancer Cells

**DOI:** 10.3390/polym11101572

**Published:** 2019-09-27

**Authors:** Brenda Gibbens-Bandala, Enrique Morales-Avila, Guillermina Ferro-Flores, Clara Santos-Cuevas, Myrna Luna-Gutiérrez, Gerardo Ramírez-Nava, Blanca Ocampo-García

**Affiliations:** 1 Departamento de Materiales Radiactivos, Instituto Nacional de Investigaciones Nucleares, Carretera México-Toluca S/N, Ocoyoacac, Estado de México 52750, Mexico; bren_gbb@hotmail.com (B.G.-B.); ferro_flores@yahoo.com.mx (G.F.-F.); clara_letici@yahoo.com.mx (C.S.-C.); myrna.luna@inin.gob.mx (M.L.-G.); gerjul5420@hotmail.com (G.R.-N.); 2 Facultad de Química, Universidad Autónoma del Estado de México, Paseo Tollocan S/N, Toluca, Estado de México 50180, Mexico; enrimorafm@yahoo.com.mx; 3 Departamento de Bioprocesos, UPIBI-Instituto Politécnico Nacional, Ciudad de México 07340, Mexico

**Keywords:** polymeric nanosystems, dendrimers, radiotherapy, GRPr, paclitaxel, lutetium-177

## Abstract

The peptide-receptor radionuclide therapy (PRRT) is a successful approach for selectively delivering radiation within tumor sites through specific recognition of radiolabeled peptides by overexpressed receptors on cancer cell surfaces. The efficacy of PRRT could be improved by using polymeric radio- and drug- therapy nanoparticles for a concomitant therapeutic effect on malignant cells. This research aimed to prepare and evaluate, a novel drug and radiation delivery nanosystem based on the ^177^Lu-labeled polyamidoamine (PAMAM) dendrimer (DN) loaded with paclitaxel (PTX) and functionalized on the surface with the Lys^1^Lys^3^(DOTA)-bombesin (BN) peptide for specific targeting to gastrin-releasing peptide receptors (GRPr) overexpressed on breast cancer cells. DN was first conjugated covalently to BN and DOTA (chemical moiety for lutetium-177 complexing) and subsequently loaded with PTX. The characterization by microscopic and spectroscopic techniques, in-vitro drug delivery tests as well as in in-vitro and in-vivo cellular uptake of ^177^Lu-DOTA-DN(PTX)-BN by T47D breast cancer cells (GRPr-positive), indicated the formation of an improved delivery nanosystem with target-specific recognition by GRPr. Results of the ^177^Lu-DOTA-DN(PTX)-BN effect on T47D cell viability (1.3%, compared with 10.9% of ^177^Lu-DOTA-DN-BN and 14.0% of DOTA-DN-(PTX)-BN) demonstrated the concomitant radiotherapeutic and chemotherapeutic properties of the polymeric nanosystem as a potential agent for the treatment of GRPr-positive tumors.

## 1. Introduction

In cases of metastatic or inoperable malignant diseases, the peptide-receptor radionuclide therapy (PRRT) is a viable option for selective delivering of radiation in tumors through specific recognition of radiolabeled peptides by receptors overexpressed on cancer cell surfaces [1].

Beta emitting radionuclides, such as lutetium-177 (*T*_1/2_ = 6.7 d, βmax emission of 497 keV, *γ*-emission of 113 and 208 keV), are widely used in PRRT due to their relatively long-range in tissues (12 mm), which allows a cross-fire effect with the surrounding cells into the tumor.

Gastrin-releasing peptide receptors (GRPr) are overexpressed in breast, prostate, gastric, colon, and pancreatic tumors [2]. Various GRP analogs (e.g., bombesin or bombesin-modified peptides) labeled with Ga-68, Y-90, In-111, and Lu-177 have demonstrated a high in-vitro and in-vivo affinity for GRPr [3,4,5].

Among various approaches, the use of polymeric nanoparticles in cancer treatment has gained significant importance. Various drug delivery and drug targeting systems such as synthetic polymers, microcapsules, liposomes, and dendrimers, are now either already approved for clinical use or under development. The nanoparticles designed as drug delivery systems can improve the bioavailability and selective accumulation of drugs at the pathological site, overcoming the challenges faced by potent anticancer medicines related to the systemic side effects and multi-drug resistance [6].

Dendrimers are hyperbranched polymers with the distinctive 3D molecular arrangement, well-defined structure, and homogeneous composition. The high level of control over dendrimers structure and their multifunctional properties makes them attractive for drug delivery applications as well as nano-radiopharmaceutical devices. The functional groups in the outermost part of the dendrimer allow attaching moieties that can actively target specific cell receptors. A promising property of dendrimers in cancer diagnosis and treatment is the multimeric nature, which would potentially produce multivalent effects [7,8,9].

Polyamidoamine (PAMAM) dendrimers are described as carriers for anticancer drugs and can be easily modified at the terminal groups by to improve their specific targeting to tumor cells [10]. Paclitaxel (PTX) is one of the best anticancer drugs and is active against a wide spectrum of cancers. However, the major limitation in the clinical use of paclitaxel is its low solubility in water and most pharmaceutical grade solvents.

The efficacy of PRRT could be improved by using a polymeric radio—and drug—therapy nanosystem for a concomitant therapeutic effect on malignant cells. In this context, the aim of this research was to prepare and evaluate a novel drug and radiation delivery nanosystem based on the ^177^Lu-labeled PAMAM dendrimer (DN) loaded with PTX and functionalized on the surface with the Lys^1^Lys^3^(DOTA)-bombesin (BN) peptide for specific targeting to gastrin-releasing peptide receptors (GRPr) overexpressed on breast cancer cells.

## 2. Materials

PAMAM dendrimer (DN) (ethylenediamine core, generation 4.0 solution 10 wt% in methanol) and Paclitaxel (PTX) were supplied by Sigma-Aldrich Chemical Co. (St. Louis, Missouri, USA). Bifunctional chelating agent S-2-(4-isothiocyanatobenzyl)-1,4,7,10-tetraazacyclododecane acid (p-SCN-Bn-DOTA) (DOTA) was obtained from Macrocyclics (Dallas, TX, USA). Lys^1^Lys^3^(DOTA)-Bombesin (bombesin, BN) was provided by International Atomic Energy Agency (through CRP-F2264). Lutetium-177 (^177^Lu) as ^177^LuCl_3_, was supplied from ITG (Germany). Dimethylformamide (DMF), diisopropylethylamine (DIPEA), 2-(1H-7-azabenzotriazol-1-yl)-1.1.3.3-tetramethyluroniumhexafluorophosphate (HATU), and all other reagents were analytical grades. 2,3-bis-(2-methoxy-4-nitro-5-sulfophenyl)-2H-tetrazolium-5-carboxanilide) (XTT) was obtained from Roche Diagnostics (Indianapolis, IN. USA). The T47-D cell line was obtained from ATCC (Atlanta, GA, USA).

## 3. Methods

### 3.1. Synthesis of DOTA-DN and DOTA-DN-BN

**Figure 1:**
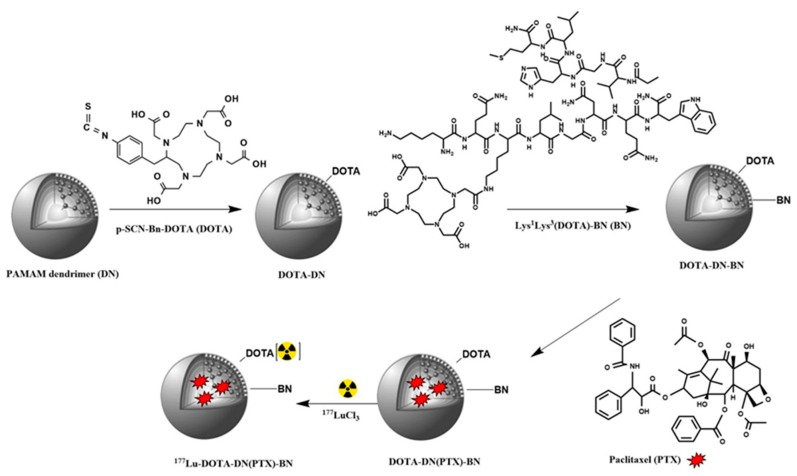
Schematic illustration of ^177^Lu-DOTA-DN(PTX)-BN preparation.

DOTA-DN was prepared according to the procedure described by Mendoza-Nava [11]. Briefly, PAMAM dendrimer methanol free (0.2859 µmol) and DOTA (4.3605 µmol) were dissolved in bicarbonate buffer (0.2 M, pH 9.5) and incubated at 37 °C during 1 h. Then, the DOTA-DN was purified by repetitive aqueous-washing until complete elimination of bicarbonate buffer (Ultra centrifugal filters, MWCO 3000 Da, Millipore, 2500 g, 30 min). Finally, the product was lyophilized (Figure 1).

In order to achieve carboxylate groups activation from Lys^1^Lys^3^(DOTA)-bombesin, the peptide (0.5 µmol) was incubated with HATU (HATU 1.3 µmol, DIPEA 19.1 µmol, and DMF as solvent) at room temperature for 15 min. The DOTA-DN (4.5 mg in bicarbonate buffer) was posteriorly added to the solution with active peptide. The mixture was then incubated during 1.5 h at room temperature. The obtained DOTA-DN-BN was purified and dried under vacuum. The Lys^1^Lys^3^(DOTA)-bombesin peptide was used as the biomolecule to reach the GRP receptor, where DOTA was used as the linker to form amide bounds with dendrimer. Since the carboxylate groups were activated and bound to primary amine, there are not enough chemical groups available to chelate Lu-177 in a stable coordination sphere at neutral pH.

For the preparation of Paclitaxel-loaded DN, a fraction of aqueous solution containing 5 mg of DOTA-DN-BN was mixed with Paclitaxel (1 mg, 1.17 µmol in methanol). The final mixture was stirred for 24 h at room temperature. The obtained PTX-loaded dendrimers were purified by ultracentrifugation and the pellet dried under vacuum to remove any non-incorporated PTX.

### 3.2. Radiolabeling of Dendrimeric Systems

Conjugated dendrimers were radiolabeled through bifunctional chelator DOTA with ^177^Lu. Radiolabeling was carried out by adding a ^177^LuCl_3_ solution (18.5 MBq in 10 μL) to DOTA-DN, DOTA-DN-BN and DOTA-DN(PTX)-BN (500 μL of 1 mg/mL in 1M acetate buffer, pH 5.0) and incubating each solution at 37 °C for 1 h.

Radiochemical purity analyses were performed by ultracentrifugation and instant thin-layer chromatography on silica gel (ITLC-SG, Gelman Sciences), using NaCl 0.9%/HCl 0.02% as mobile phase.

### 3.3. Physicochemical Characterization

#### 3.3.1. Transmission Electron Microscopy (TEM)

Dendrimers were analyzed by TEM in JEOL JEM 2010 HT microscope operating at 200 kV to observe the two-dimensional, relative size, distribution, and morphology. A drop of the aqueous product was evaporated for analysis on a carbon-coated TEM copper grid. Approximately 1000 nanoparticles from 11 TEM micrographs were analyzed.

#### 3.3.2. Fourier Transform Infrared Spectroscopy (FT-IR)

The FT-IR measurements were performed in transmission mode using a Perkin Elmer System 2000 spectrometer with the attenuated total reflection platform (Pike Technologies; Madison, WI, USA). The spectra were all acquired from 50 scans at a 0.4 cm^−1^ from 400 to 4000 cm^−1^.

#### 3.3.3. Entrapment Efficiency

Paclitaxel analysis was carried out by HPLC (µBondapak^®^ C18 10 µm 125A°, 3.9 × 150 mm Column as stationary phase, and acetonitrile: water (60:40 *v/v*) as the mobile phase, flow rate of 1 mL/min, run time of 5 min). PTX was measured at 227 nm on a Waters Empower system using a PTX calibration curve (*R*^2^ 0.997, 6.0–300.0 µg/mL). The experiments were conducted in triplicate.

The amount of encapsulated paclitaxel was indirectly measured by subtracting the amount of non-encapsulated paclitaxel in the waste solution obtained from the amount of paclitaxel used to prepare the dendrimers. The encapsulation efficiency (*EE*%) was calculated as:

(1) $$EE\;\left(\%\right)=\frac{Weight\;of\;PTX\;loaded}{Weight\;of\;PTX\;input}\;\times \;100$$

#### 3.3.4. Conjugation Efficiency of DOTA and Bombesin

Conjugation efficiency was determined by measuring free DOTA or free Bombesin in the respective filtered solution via reverse-phase HPLC on a Waters Empower system using a C18 column (µBondapak^®^ C18 10 µm 125A°, 3.9 × 300 mm Column) as a stationary phase and a gradient of water/acetonitrile containing 0.1% TFA from 95/5 (*v/v*) to 20/80 (*v/v*) as mobile phase (flow rate of 1 mL/min and time run 30 min). Bombesin standard curve (R^2^ 0.999, 0.07–2.2 mg/mL) was used in this analysis.

#### 3.3.5. In Vitro PTX Release

For the in vitro drug release studies, a solution of phosphate-buffered saline (PBS, pH 7.4 or 5.3) containing 0.05% Tween-20 was used. Briefly, 10mg of DOTA-DN(PTX)-BN was dispersed in 1mL of release medium and placed in a dialysis membrane (MWCO 30,000 Da). The dialysis bag was closed and immersed into a flask containing PBS (10 mL) at 37 °C and continuously stirring at 110 rpm. At specific time intervals, an aliquot of release medium was collected, and replaced with fresh PBS. Released paclitaxel amount was measured by HPLC based on the previously described methodology.

#### 3.3.6. Radiolabeling Stability

For the stability evaluation of the system in serum, ^177^Lu-DOTA-DN(PTX)-BN and fresh human serum (dilution 1:10) were incubated at 37 °C. At given time points, an aliquot was taken and analyzed by radio-HPLC using a size-exclusion HPLC column (ProteinPak 300SW Waters; Milford, MA, USA) as stationary phase and 0.01M PBS as mobile phase (1 mL/min flow rate, and time run 30 min).

### 3.4. In Vitro Studies

#### 3.4.1. Cell Line

T47D Breast Epithelial Ductal Carcinoma cells positive to GRPr were grown at 37 °C in an atmosphere of 5% CO_2_ and 85% humidity in RPMI-1640 medium supplemented with 10% newborn calf serum and antibiotics (100 μg/mL streptomycin, 100 U/mL penicillin).

#### 3.4.2. Cellular Uptake

For the measurement of the nanoradiopharmaceutical fraction on the cell membrane and the internalized fraction to the cytoplasm, T47D cells were harvested to be seeded in 24-well tissue culture plates (1 × 10^5^ cell/well, 0.5 mL) to allow adherence. After 24 h, the RPMI medium was removed, and the cells were incubated with 118 kBq (per well) of the following treatment: (^177^Lu-DOTA-DN, ^177^Lu-DOTA-DN-BN and ^177^Lu-DOTA-DN(PTX)-BN) for 1 h at 37 °C. Then, each well was rinsed with PBS (2×) to eliminate the treatment. The plate was then incubated twice with 500 µL of glycine buffer (50 mM, pH 2.8) by 5 min at room temperature to obtain the fraction of cell uptake (radioactivity in the membrane). The cells were washed and incubated 5 min at room temperature with 500 µL of 1 M NaOH to evaluate the nanosystem internalization. Radioactivity was measured in a crystal scintillation well-type detector (Auto In-v-Tron 4010, NML Inc., Milwaukee, WI, USA). The uptake percentage was then calculated, considering the initial activity of each treatment as 100%. Non-specific binding was determined in parallel, in the presence of 175 nmol of Bombesin (500 times higher than the added concentration of treatments) to partially block the GRPr present in T47D cells. Additionaly, the saturation assay was performed as previously reported [12].

#### 3.4.3. Cytotoxic Effect on T47D Cells

T47D cells were seeded in 96-well microtiter plate (1 × 10^4^ cells/well) and incubated 24 h to allow adherence. The viability after exposure to 0.45 MBq of ^177^Lu-DOTA-DN-BN, ^177^Lu-DOTA-DN(PTX)-BN and ^177^LuCl_3_ or 4µg of free PTX, DOTA-DN(PTX)-BN (in terms of paclitaxel amount) or equimolar concentration of DOTA-DN-BN, was evaluated at different time points (7 days, 37 °C, 5%°O_2_ and 85% humidity). The cytotoxic activity was measured using a Cell Proliferation Assay (XTT) kit, according to the manufacturer’s protocol (Roche Diagnostics GmbH, Mannheim, Germany).

#### 3.4.4. Radiation-Absorbed Doses to the T47D Cell Nucleus

For estimation of the radiation-absorbed doses to the T47D cell nucleus, the following equation was used:

(2) $$D_{N\leftarrow Source}=\left(N_{M}\;\times DF_{N\leftarrow M}\right)+\left(N_{C}\;\times DF_{N\leftarrow C}\right)$$

where *D_N__←Source_* represents the mean absorbed dose to the nucleus from source regions (membrane-M- and cytoplasm-C-) and *N_M_* and *N_C_* are the total number of nuclear disintegrations that occurred in the membrane and cytoplasm, respectively. *DF_N__←M_* and *DF_N__←C_* denote the dose factors specific for ^177^Lu, from membrane and cytoplasm regions to the nucleus configuration. The dose factor geometries were obtained from the S values reported by Goddu and Budinger (cell radius = 10 µm, nucleus radius = 5 µm) [13].

#### 3.4.5. In-Vitro Cell Treatment

The required radioactivity of ^177^Lu-DOTA-DN(PTX)-BN to produce approximately the standard breast post-surgical radiotherapy (50 Gy) [14], was calculated by using the experimental biokinetic model fitted in Section 3.4.4. Half of this concentration was also prepared to evaluate the response related to PTX alone or ^177^Lu/PTX. Therefore, 32.2 Bq and 16.1 Bq per cell was deposited on a 96-well plate containing 10,000 T47D cells per well. The identical unlabelled concentration of DOTA-DN(PTX)-BN was also evaluated at each level (*n* = 6).

### 3.5. In Vivo Studies

In vivo studies in mice were carried out according to the Official Mexican Norm 062-ZOO-1999. Athymic mice, 6–7 weeks old and 18–20 g weight, were kept in sterile cages with sterile wood-shaving beds, constant temperature, humidity, and 12:12 light: dark periods.

*Tumor Induction*. Animals (*n* = 4) received a subcutaneous inoculation (upper backsubcutaneous tumor model-) of 1 × 10^6^ T47D cancer cells suspended in 0.1 mL of phosphate-buffered saline. 2.5 weeks after inoculation the animals were used for in vivo studies. One mouse was randomly selected for micro-SPECT/CT imaging, and a group of three mice was used to perform biodistribution studies.

#### 3.5.1. SPECT-CT Imaging

To verify the in vivo ^177^Lu-DOTA-DN(PTX)-BN retention in T47D induced tumors, micro-SPECT/CT images were acquired (Albira, ONCOVISION; Gem Imaging S.A., Valencia, Spain) 1.5 h, 9 h, 10 h, 24 h, 120 h after intratumoral administration of 9.25 MBq of the nanoradiopharmaceutical. The mouse, under 2% isoflurane anesthesia, was placed in the prone position and imaging was performed. The micro-SPECT field of view was 60 mm; a symmetric 20% window was set at 208 keV, and pinhole collimators were used to acquire a three-dimensional SPECT image with a total of 64 projections of 30 s each over 360°. The image was reconstructed using the ordered-subset expectation-maximization algorithm with the standard mode parameter, as provided by the manufacturer. CT parameters were 35 kV positive voltage, 700 µA current, and 600 micro-CT projections.

#### 3.5.2. Biodistribution

The subcutaneous tumor model animals (*n* = 3) received 9.25 MBq of the radiolabeled system (^177^Lu-DOTA-DN(PTX)-BN). After 120 h post-injection, the mice were euthanized, and the blood and main organs were removed. The radioactivity accumulated in organs was measured in a calibrated Na(Tl) detector and expressed as %ID/g (percentage of the injected dose per gram of tissue) or %ID/organ (percentage of the injected dose per organ).

## 4. Results and Discussion

PAMAM dendrimers have extensively been reported as drug delivery systems for carrying therapeutic and diagnostic agents, but their capacity for concomitant applications of chemotherapy (Paclitaxel) and radiotherapy (^177^Lu) in a target-specific multifunctional platform has not been reported.

PAMAM dendrimer generation 4.0 contains 64 terminal amine groups available for firstly grafting with p-SCN-Bn-DOTA, via the irreversible formation of thiourea derivative. Bombesin conjugation was attained via one carboxyl lateral arm from the DOTA macrocycle, which was linked to the PAMAM amine group. The calculated DN:DOTA and DN:Bombesin molar ratio was 1:15 and 1:3, respectively.

A variety of delivery systems of PTX have been reported, including entrapment of the molecule [15,16,17], or conjugation to the surface [18,19,20]. In this research, the dendrimeric system allowed 97.72 ± 0.26% of PTX encapsulation.

**Figure 2:**
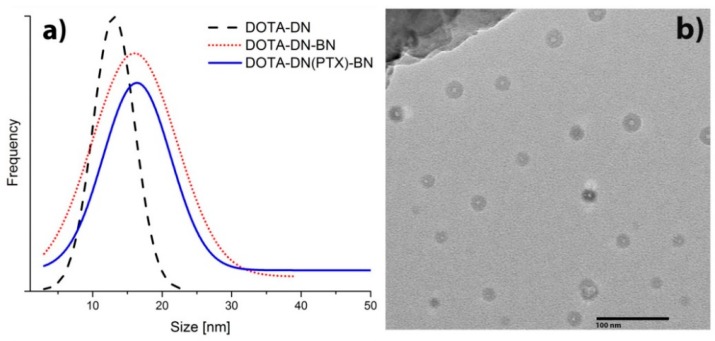
(**a**) Size distribution of the different nanosystems (**b**) TEM of DOTA-DN(PTX)-BN.

As shown in Figure 2, microscopic transmission electron analysis (TEM) revealed well-defined dendritic structures (Figure 2b). The mean particle size of DOTA-DN was 13.05 nm, 16.01 nm for DOTA-DN-BN, and 16.37 nm in the case of DOTA-DN(PTX)-BN. The BN grafting, influenced on increasing the particle size (*p* < 0.05). Also, the population width distribution increased with bombesin and paclitaxel incorporation.

### 4.1. Infra-Red Spectroscopy

FT-IR analysis of the DOTA-DN(PTX)-BN dendrimeric system showed some characteristic bands of its components (Dendrimer, DOTA, bombesin, and paclitaxel) which changed in intensity and position because of the conjugation reactions.

**Figure 3:**
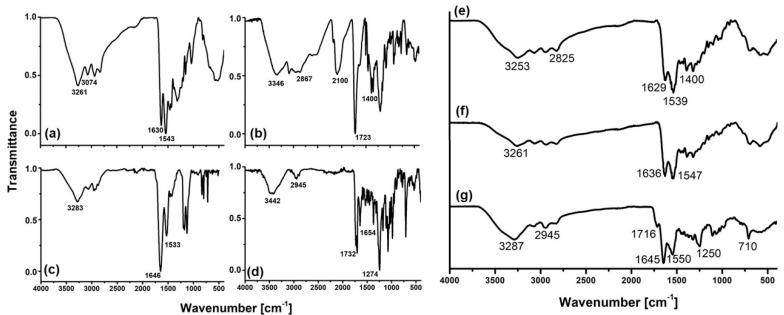
The FT-IR spectrum of (**a**) PAMAM Dendrimer (DN), (**b**) pSCN-Bn-DOTA (DOTA), (**c**) Lys^1^Lys^3^(DOTA)-BN (BN), (**d**) Paclitaxel (PTX), (**e**) DOTA-DN, (**f**) DOTA-DN-BN, (**g**) DOTA-DN(PTX)-BN.

The IR-spectrum of pure PAMAM G4.0 (Figure 3a) showed bands at 3261 cm^−1^ and 3074 cm^−1^ attributed to N-H stretch vibrations of amine groups. The bands at 2935 cm^−1^ and 2831 cm^−1^ correspond to aliphatic C-H stretches. The amide carbonyl absorption (HNC = O) from the PAMAM dendrimer was observed at 1630 cm^−1^, while at 1543 cm^−1^, the amide N-C stretching was identified [21,22,23,24].

The p-SCN-Bn-DOTA IR-spectrum (Figure 3b) showed a well-defined band at 2100 cm^−1^, which was assigned to the isothiocyanate motif vibration. At 2960–2867 cm^−1^ and 1400 cm^−1^ appear vibrations attributed to the C-H stretch, and at 3346 cm^−1^ the presence of amine groups were identified. At 1723 cm^−1^ the C=O stretch and at 1500 cm^−1^ the aromatic C-C stretch were also observed.

After p-SCN-DOTA conjugation to dendrimer, the DOTA-DN (Figure 3e) showed a shift compared to the main dendrimer bands in the region at 3000–2900cm^−1^. New bands in the amide I and amide II region were observed. The isothiocyanate region from the bifunctional agent was absent, which suggested a conformational and dipole change when conjugation was carried out. Amide formation involves the use of carboxylic groups, which was not observed after conjugation. The new band at 1400 cm^−1^ region indicated the presence of DOTA in the dendrimer structure.

The IR-spectrum of pure BN (Lys^1^Lys^3^(DOTA)BN) (Figure 3c) showed bands at 3283 cm^−1^, 1646 cm^−1^ and 1533 cm^−1^ assigned to (–NH)_υ_, (C=O)_υ_ (Amide I) and (–CN) (amide II) respectively, as previously reported [25].

After grafting DOTA-DN with Bombesin peptide, the infrared spectrum showed essential changes. The new product DOTA-DN-BN (Figure 3f) show significant shifts in the amine and amide region, but no new bands were observed.

The IR analysis of PTX (Figure 3d) was consistent with the previously reported [26]. Briefly, spectrum showed (–NH)_υ_ vibrations characterized by a broad and asymmetric band centered at 3442 cm^−1^, the band found at 2945 cm^−1^ was assigned to (–C-H) from asymmetric and symmetric stretching vibrations. Amide I region mainly associated with (C=O)_υ_ vibration was identified at 1732 cm^−1^ whereas (C-N)_υ_ was found at 1274 cm^−1^. Aromatic hydrocarbons were identified by characteristic absorption bands in the region near to 1645 and 1500–1400 cm^−1^ produced by carbon-carbon stretching vibrations in the aromatic ring. The bands at 1250–1000 cm^−1^ region were assigned to (C–H) in-plane bending, and finally, (C–H)_υ_ above 3000 cm^−1^ were also identified.

After PTX interaction (Figure 3g), the infrared spectrum showed bands of PTX at 1716 cm^−1^ and 710 cm^−1^ from amide I, additionally, an increase of intensity was observed in the well-defined bands at 1645 cm^−1^ and 1400 cm^−1^ from Ar(C-C) and 1250 cm^−1^ due to the contribution of amine groups. A significant increase on band intensity and change on band shape near to 3650–3150 cm^−1^ region respect to DOTA-DN-BN were identified, suggesting an increase on H-bond formation associated to the interaction of O-H and N-H groups as proton donor groups from PTX, BN, and DN.

### 4.2. In Vitro Paclitaxel Release

For evaluation of the controlled release capacity of paclitaxel from DOTA-DN(PTX)-BN, accumulative (%) drug release was determined in different analysis conditions.

**Figure 4:**
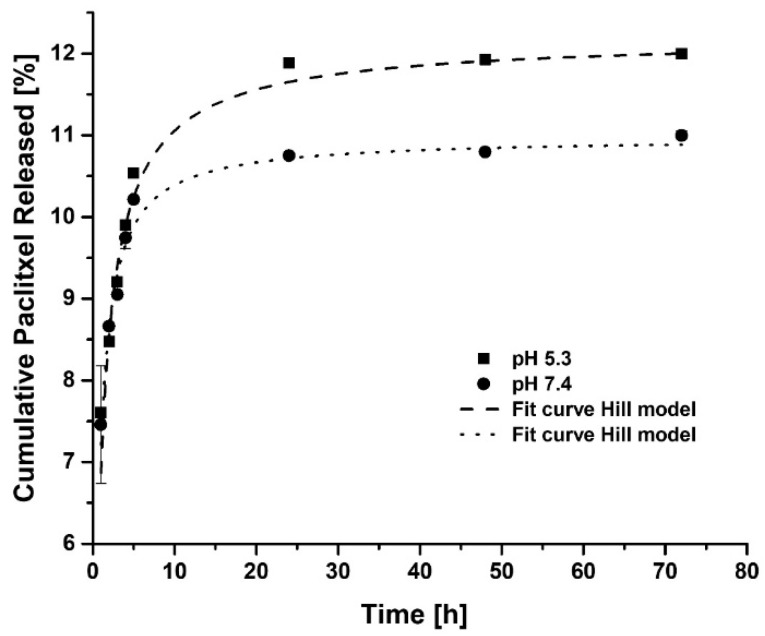
In vitro release profile of Paclitaxel from DOTA-DN(PTX)-BN system at pH 5.3 and 7.4 (mean ± SD, *n* = 3).

The release profile of PTX was prolonged. The PTX desorption was dependent on pH and resulted to be higher under acidic conditions than under neutral conditions (Figure 4, Hill Model) through disturbance and disruption of hydrogen bonding. Hence, the observed release rate of DOTA-DN(PTX)-BN at pH 5.3 is favorable for cancer therapy. Additionally, it would be expected that acidic intra-cellular conditions (intracellular lysosomal environment) improve the PTX release with the consequent increase of local drug concentration.

The relatively slow PTX-releasing rate observed in vitro could be enhanced by the in vivo high oncotic pressure in tumors, since previous reports have demonstrated that the PTX release from dendrimeric systems gradually speeds up with the osmotic pressure [27].

### 4.3. Serum Stability

^177^Lu-DOTA-DN(PTX)-BN demonstrates high stability in human serum. After 0.5, 1 and 24 h of incubation <10% of the radiopharmaceutical was bound to plasma proteins. These results suggest that ^177^Lu-DOTA-DN(PTX)-BN was not significantly catabolized in serum, and therefore has suitable metabolic stability.

### 4.4. In Vitro Studies

#### 4.4.1. Cell Uptake

**Figure 5:**
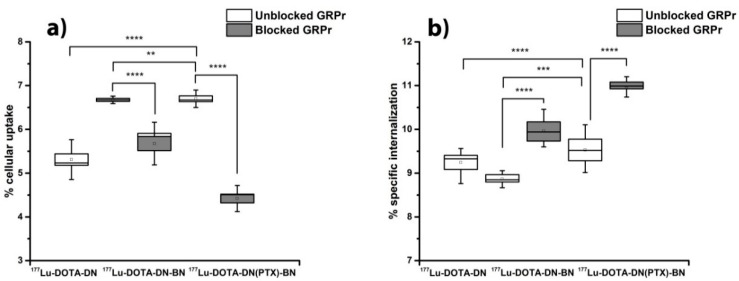
Uptake (**a**) and internalization (**b**) of ^177^Lu-DOTA-DN, ^177^Lu-DOTA-DN-BN, and ^177^Lu-DOTA-DN(PTX)-BN in T47D cells (vacuum boxes). Cells with blocked receptors were pre-incubated with excess (500 times) of Bombesin peptide (full boxes) to determine specific uptake. Whisker represents SD (*n* = 6).

In order to demonstrate the specific uptake of nanoradiopharmaceuticals via Bombesin-GRPr recognition, uptake and internalization assays in T47D cells (GRPr-positive) were carried out (Figure 5). The ANOVA (1 way) for multiple comparisons showed a statistical significant difference between ^177^Lu-DOTA-DN(PTX)-BN and ^177^Lu-DOTA-DN (*p* < 0.05) (cell uptake of 6.67% vs 5.31%, respectively), which confirmed the active uptake mechanism through the interaction Bombesin-GRPr. There was no significant difference between nanosystems containing the molecular recognition molecule (^177^Lu-DOTA-DN-BN, 6.67% and ^177^Lu-DOTA-DN(PTX)-BN, 6.70%).

Figure 5b shows the internalization of each radiolabelled treatment to the cytoplasm. This process was favored with the pre-incubation with BN and attributed to cationic surface charge conferred by BN, which promotes interaction of the nanoparticles with the membrane and therefore, increases the rate and extent of internalization [28].

When GRPr were previously blocked with a BN concentration 500 higher than the BN present at nanosystems, the uptake decreased one-fold and two-fold for ^177^Lu-DOTA-DN-BN and ^177^Lu-DOTA-DN(PTX)-BN, respectively. This result suggests that the process of specific binding between Bombesin and GRPr is taking place through an endocytic pathway in high rate for ^177^Lu-DOTA-DN(PTX)-BN rather than the non-targeted nanosystem, which could be favored by the high molecular weight conferred by PTX inside the cavities. This specific coupling provided a significant uptake and internalization (internalization of 8.86% in ^177^Lu-DOTA-DN-BN and 9.53% in ^177^Lu-DOTA-DN(PTX)-BN). However, it was evident that passive uptake was carried out by a surface phenomenon between nanoparticles and the cell surface. The ^177^Lu-DOTA-DN(PTX)-BN concentration needed to achieve the half-maximum binding at equilibrium (Kd) resulted in 32.49 ± 9.01 nM.

#### 4.4.2. Cell Viability

The surface coating has been reported to determine the cytotoxicity/biocompatibility for many nanoparticles. Particularly for PAMAM dendrimers, toxicity is mainly attributed to surface amine groups, but also dependent upon molecular weight, the number of surface amine groups, and generation of PAMAM dendrimers. Several studies have reported that after surface modification, the toxicity of PAMAM dendrimers can be reduced [29].

**Figure 6:**
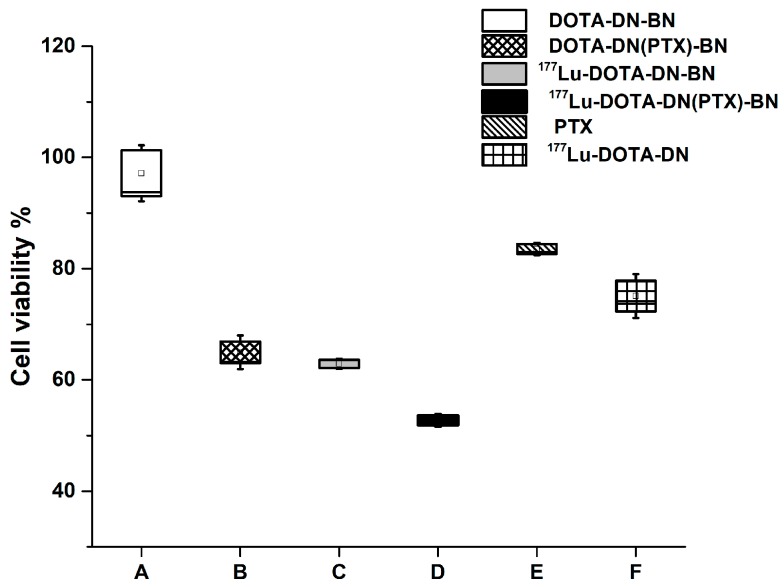
Cell viability after seven days of treatment with DOTA-DN-BN (**A**), DOTA-DN(PTX)-BN (**B**), ^177^Lu-DOTA-DN-BN (**C**), ^177^Lu-DOTA-DN(PTX)-BN (**D**), Paclitaxel (**E**), ^177^Lu-DOTA-DN (**F**).

As shown in Figure 6, the dendrimer surface modification with DOTA and Bombesin, allowed the preparation of a non-toxic system DOTA-DN-BN (97.14 ± 10.12% of viability), mainly attributed to the surface end groups [7].

Reduction on cell viability was inherent to the presence of PTX or ^177^Lu. The effect of combining ^177^Lu with PTX was the highest on reducing viability on T47D cells treated with ^177^Lu-DOTA-DN(PTX)-BN (Figure 6). At seven days post-treatment, viability percentage was only 54.15 ± 3.05% for ^177^Lu-DOTA-DN(PTX)-BN, which was significantly different (*p* < 0.05) when compared with the unlabeled system DOTA-DN(PTX)-BN (64.58 ± 6.32%), or free-PTX system ^177^Lu-DOTA-DN-BN (59.03 ± 5.48%).

As expected, there was no statistical significance between the cell uptake of DOTA-DN(PTX)-BN when compared with ^177^Lu-DOTA-DN-BN (*p* > 0.05). This therefore implies that the produced cytotoxic effect caused by ^177^Lu and PTX was at the same level.

**Figure 7:**
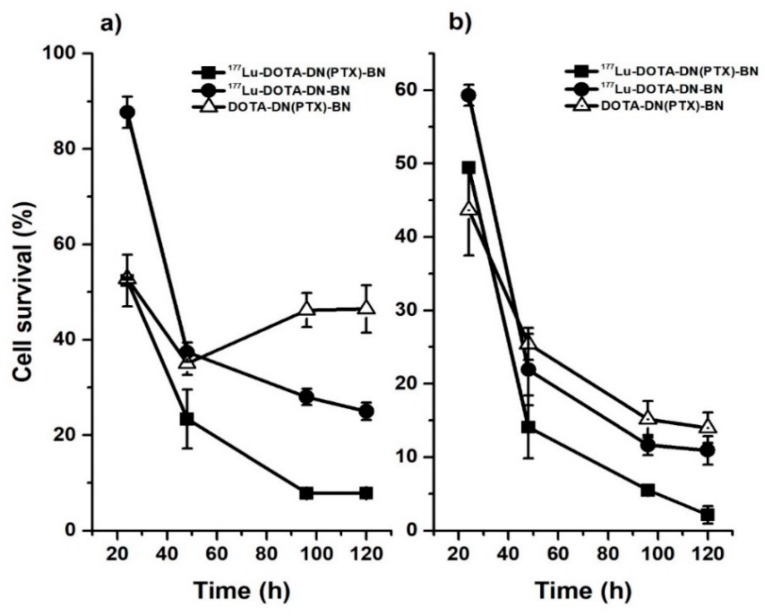
In vitro therapeutic efficacy of ^177^Lu-DOTA-DN-(PTX)-BN on T47D cell line, (**a**) cell survival fraction resulted from 16.1 MBq per cell and the equimolar concentration of the unlabeled nanosystem, (**b**) cell survival fraction resulted from 32.2 MBq per cell and the equimolar level of the unlabeled nanosystem.

The in vitro therapeutic efficacy studies confirmed that ^177^Lu-DOTA-DN-(PTX)-BN nanosystem nanoparticles are more effective than a non-radiolabeled nanoparticle. The synergistic effect of chemotherapy and radiotherapy produced by the ^177^Lu-DOTA-DN-(PTX)-BN nanosystem was observed when doses of PTX = 26.79 µM and radiation-absorbed doses of 122 Gy (delivered by 32.19 Bq/cell) were used (Table 1). As can be observed in Figure 7, the cytotoxic effect for the radiolabeled nanosystem was observed between 24 h and 48 h (highest slope). The effect was statistically significant after 48, 96, and 120 h. At 120 h of treatment, the total delivered radiation dose was 122 Gy, and the concentration of PTX was 26.79 µM.

Nanosystems containing and delivering both, chemo—and radiotherapy are more effective than nanoparticles containing only PTX. When T47D breast cells were treated with the targeted nanosystem providing only PTX (DOTA-DN-(PTX)-BN) at 13.40 µM, the cell viability increased after 48 h (Figure 7a). This behavior was attributed to the DNA repair rate. The concentration of delivered PTX was unsuccessful in producing disrupting of microtubules. After 120 h of treatment with DOTA-DN-(PTX)-BN, the cell viability was 14.00 ± 2.06%. The viability of cells treated with ^177^Lu-DOTA-DN-BN was 10.93 ± 1.17%, and the treatment of cells with ^177^Lu-DOTA-DN-(PTX)-BN resulted in the lowest viability of 1.32 ± 0.71%. These results demonstrated that the combination of PTX and Lu-177 in the same nanoprobe was more effective than PTX or Lu-177 alone.

#### 4.4.3. In Vivo Studies 

The ^177^Lu-DOTA-DN-(PTX)-BN nanosystem showed high retention in the tumor volume. No significant leakage was observed after 120 h associated with the specific receptor binding [11], which suggest that this nano-approach has a strong potential to be used for the treatment of solid tumor over-expressing GRPr. (Ethical Approval number: NOM-062-ZOO-1999 and approval date: 7 March 2018 for animal experiments).

**Figure 8:**
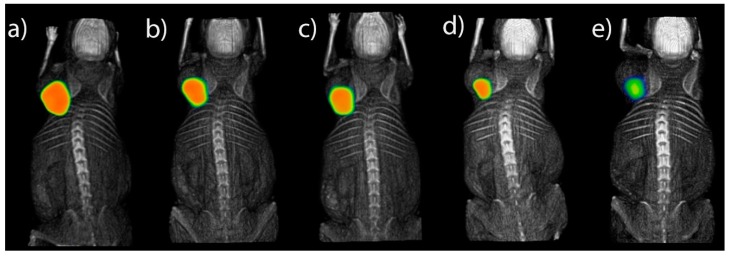
Subcutaneous tumoral model. Intratumoral administration of ^177^Lu-DOTA-DN(PTX)-BN after (**a**) 1.5 h, (**b**) 9 h, (**c**) 10 h, (**d**) 24 h, (**e**) 120 h.

Figure 8 shows micro-SPECT images of a mouse with a T47D subcutaneous tumor model. The tumor-to-blood ratio reveals suitable contrast, with a significant accumulation of ^177^Lu-DOTA-DN(PTX)-BN in tumor tissue after (a) 1.5 h, (b) 9 h, (c) 10 h, (d) 24 h, and (e) 120 h. The ^177^Lu-DOTA-DN(PTX)-BN nanosystem remains into the tumor delivering both, targeted radiotherapy and PTX-chemotherapy.

The measured volume of the tumor at the beginning of the imaging studies (1.5 h) was 0.32 cm^3^. The biokinetic model based on the standard uptake value obtained at each time point, was adjusted to a function in OLINDA/EXM. The estimated absorbed radiation dose was 3.01 Gy/MBq at infinite time (Table 2). Therefore, to produce a tumoral radiation absorbed dose of 50 Gy in the murine model (which is usually applied in radiotherapy treatments to breast cancer patients), 16.61 MBq should be administered.

The radiopharmaceutical was cleared from the tumor with three components: 7.7% of the total dose (total activity of the administered radiopharmaceutical) was removed quickly with an effective half-life (*Te*) of 1.65 h, 8.6% with a *Te* of 86 h and 0.57% with prolonged *Te* of 173 h (Table 2).

The ^177^Lu-DOTA-DN(PTX)-BN remained into the tumor delivering both, targeted radiotherapy and PTX-chemotherapy. The final tumor size was reduced 15.6% and the volume at 120 h was 0.27 cm^3^.

**Figure 9:**
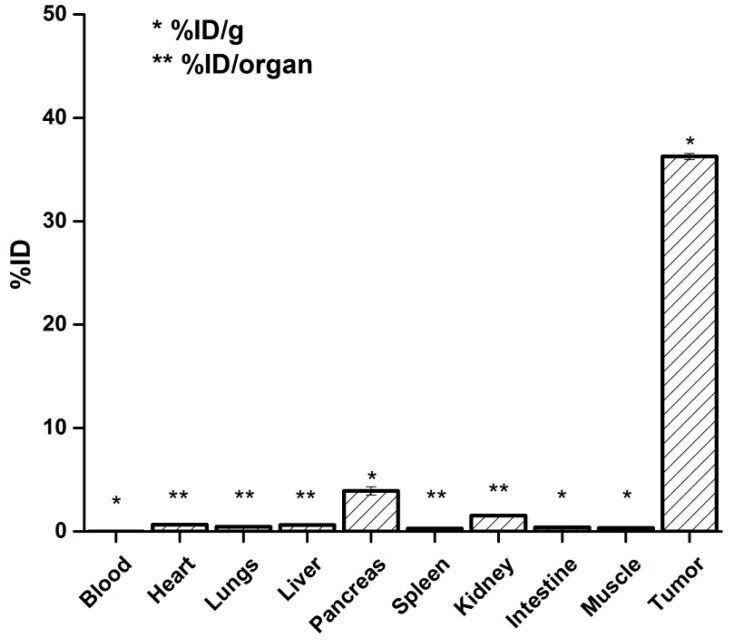
Biodistribution of ^177^Lu-DOTA-DN(PTX)-BN 72h post intratumoral administration.

Nano-radiopharmaceuticals have to be injected intratumorally or peritumorally due to the nanoparticles nature since they accumulate significantly in the reticuloendothelial system when the intravenous administration is used. The biodistribution of ^177^Lu-DOTA-DN(PTX)-BN after 120 h (Figure 9) demonstrated that 36.25% of the radioactivity remained into the tumor, with only a 3.93% in the pancreas and 1.53% in kidney, which are organs widely reported to express GRPr [30].

## 5. Conclusions

In this research, targeted polymeric nanoparticles designed to produce a simultaneous effect of radiotherapy and chemotherapy were successfully prepared. The ^177^Lu-DOTA-DN(PTX)-BN nanoparticles exhibited high affinity towards GRP receptors, which enabled them to selectively and concomitantly deliver ^177^Lu as the radiotherapeutic agent and PTX as the chemotherapeutic component. ^177^Lu-DOTA-DN(PTX)-BN showed suitable characteristics for combined targeted therapy applications in GRPr-positive tumors.

## Figures and Tables

**Table 1:** Biokinetic model and radiation absorbed doses produced by Bq/cell of ^177^Lu- DOTA-DN-(PTX)-BN to the T47D cancer cell nuclei within 168 h.

^177^Lu- DOTA-DN-(PTX)-BN Cellular Location	Biokinetic Model A(t)	$N=\overset{t=168h}{\underset{t=0}{\int }}A\left(t\right)dt$	Dose (Gy/Bq)	Total Dose to Cell Nuclei (Gy/Bq)
				4.23
Membrane	$A\left(t\right)=6.190e^{-0.014t}+0.243e^{-0.008t}+0.049e^{-0.008t}$	15012	1.12	
Cytoplasm	$A\left(t\right)=8.580e^{-0.014t}+0.345e^{-0.008t}$	20700	3.11	

**Table 2:** Biokinetic model and radiation absorbed dose produced by MBq of ^177^Lu-BN-PLGA(PTX) to the T47D tumor (intratumoral administration).

Biokinetic Model A(t)	$N=\overset{t=\infty }{\underset{t=0}{\int }}A\left(t\right)dt$	Dose (Gy/MBq)
$A\left(t\right)=7.70e^{-0.418t}+8.65e^{-0.008t}+0.57e^{-0.004t}$	11.73	3.01
